# Stereotactic Radiosurgery for Intracranial Breast Metastases: A Systematic Review and Meta-Analysis

**DOI:** 10.3390/cancers16203551

**Published:** 2024-10-21

**Authors:** Neil D. Almeida, Cathleen Kuo, Tyler V. Schrand, Julia Rupp, Venkatesh S. Madhugiri, Victor Goulenko, Rohil Shekher, Chirag Shah, Dheerendra Prasad

**Affiliations:** 1 Department of Radiation Medicine, Roswell Park Comprehensive Cancer Center, Buffalo, NY 14203, USA; neil.almeida@roswellpark.org (N.D.A.); ckuo9@buffalo.edu (C.K.); victor.goulenko@roswellpark.org (V.G.); rohil.shekher@roswellpark.org (R.S.); 2 Jacobs School of Medicine and Biomedical Sciences, Buffalo, NY 14203, USA; juliarup@buffalo.edu; 3 Department of Biochemistry and Molecular Biology, Tulane University School of Medicine, New Orleans, LA 70112, USA; tschrand@tulane.edu; 4 Department of Radiation Oncology, Taussig Cancer Institute, Cleveland Clinic, Cleveland, OH 44106, USA; shahc4@ccf.org; 5 Department of Neurosurgery, Roswell Park Comprehensive Cancer Center, Buffalo, NY 14203, USA

**Keywords:** stereotactic radiosurgery, breast cancer, intracranial metastasis, Gamma Knife radiosurgery

## Abstract

**Simple Summary:**

Breast cancer has a high predilection for intracranial metastases, second only to lung cancer. The development of intracranial metastases adds significant morbidity and mortality, with the treatment paradigm evolving greatly over the past two decades. The emergence of stereotactic radiosurgery has allowed for the delivery of a highly conformal tumoricidal dose, providing optimal local control while minimizing neurocognitive impairment. Although SRS has demonstrated effectiveness in treating brain metastases, there is significant variability in the methodologies and patient populations across studies focusing on its application for intracranial metastases originating from breast cancer. We conducted a meta-analysis to better understand the impact of stereotactic radiosurgery on the management of intracranial metastases from breast cancer.

**Abstract:**

Background/Objectives: To determine the impact of stereotactic radiosurgery on outcomes of metastatic breast cancer with intracranial metastases. Methods: We systematically searched the PubMed and EMBASE databases for studies published between 1 January 1990 and 1 August 2024. Primary research articles evaluating the outcomes of stereotactic radiosurgery on intracranial metastases from breast cancer were included. Adverse events were defined as leptomeningeal disease, radiation necrosis, seizure, and headache. The pooled estimate was calculated using the DerSimonian and Laird approach. Results: Sixteen studies encompassing 1228 patients met the inclusion criteria. Our analysis revealed a median survival duration of 13.1 ± 3.8 months and a pooled 1-year overall survival rate of 53.1% after SRS treatment. There was a 29% local recurrence rate at 1 year and a 35% overall distant recurrence rate. In addition, our analysis found a relatively low rate of acute adverse events at 15.5%. Conclusions: SRS demonstrates promising efficacy and safety in managing intracranial metastases from breast cancer, with a favorable toxicity profile.

## 1. Introduction

Breast cancer is currently the most commonly diagnosed non-cutaneous cancer worldwide, with a reported incidence of 2.26 million cases worldwide in 2020 [1,2]. For patients with metastatic breast cancer, brain metastases are one of the most common sites, second only to lung cancer [3,4]. The incidence of developing brain metastases from breast cancer at the time of diagnosis is between 7 and 16% and contributes significantly to morbidity and mortality [3,4,5,6].

Breast cancer can be subclassified based on its tumor receptor type and molecular expression into Luminal A or B, HER2+, or triple-negative. Tumor receptor status has a notable influence on the risk of development of brain metastases in breast cancer patients. Prior studies have demonstrated patients with human epidermal growth factor receptor 2 positive (HER2+) and triple-negative breast cancer (TNBC) have an increased risk of developing brain metastases [4,5,7].

Radiation therapy management of brain metastases has traditionally focused on whole-brain radiotherapy (WBRT) [8]. However, neurocognitive impairment is a significant side effect of WBRT, with patients exhibiting significant decreases in neuropsychological test scores after WBRT treatment [9,10]. Thus, stereotactic radiosurgery (SRS) has emerged as a promising treatment for the isolated treatment of metastases, or in addition to WBRT, as it has been associated with reduced levels of cognitive decline and improved quality of life compared to WBRT [3,11]. In addition to the cognitive benefit, SRS typically allows for the delivery of a high dose of conformal radiation, which has improved local control relative to WBRT [12]. Therefore, given the minimal invasiveness and improvement in cognitive function, SRS alone is deemed effective in patients with up to ten brain metastases.

Although SRS has demonstrated effectiveness in treating brain metastases, there is significant variability in the methodologies and patient populations across studies focusing on its application for intracranial metastases originating from breast cancer. This inconsistency complicates the identification of key risk factors influencing patient outcomes and guidelines for appropriate clinical utilization. To address this issue, we conducted a systematic review and meta-analysis to evaluate the existing evidence on the impact of SRS for treating intracranial metastases in patients with breast cancer.

## 2. Materials and Methods

This study was conducted in accordance with the Preferred Reporting Items for Systematic Reviews and Metanalyses (PRISMA) guidelines [13]. Review and approval were not required by our institutional review board since this study did not involve human subjects.

### 2.1. Search Strategy

We performed an extensive literature search utilizing PubMed (National Library of Medicine) and EMBASE (Elsevier, Amsterdam, The Netherlands) using a multitude of key search terms from our patient population of interest including breast cancer, breast tumors, CNS metastasis, intracranial metastasis, stereotactic radiosurgery, radiosurgery, Gamma Knife/GKRS. The time frame for the query was 1 January 1990, to 1 August 2024, and the search was limited to English language publications and human subjects.

### 2.2. Selection Criteria

Two authors (CK and JR) performed initial screening of all original article titles and abstracts to ascertain eligibility after removal of duplicates in EndNote X9 (Clarivate, London, UK). Uncertain cases were resolved upon discussion with a third author (NA). Original research articles were included in the analysis if they met the following criteria: (1) published in English; (2) focused on adult patients (>18 years) with metastatic breast cancer; (3) involved patients with one or more intracranial metastases treated with stereotactic radiosurgery.

**Figure 1:**
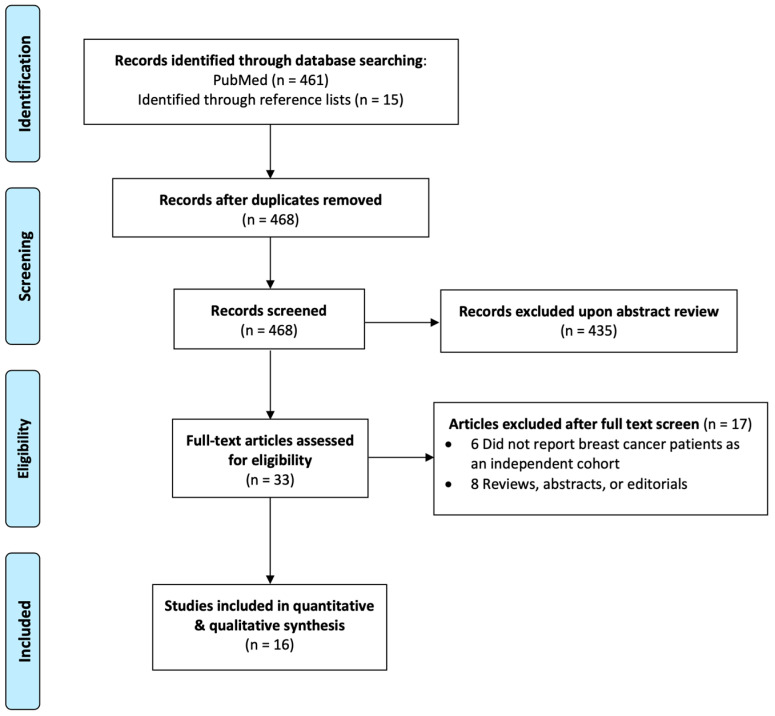
Study selection flow chart.

In the situation where two studies were reported by the same group or authors with identical patient cohorts, we included the more robust analysis. Exclusion criteria applied to all selected studies were (1) review articles, conference abstracts, or meta-analyses; (2) studies involving animals or cell lines; and (3) studies including patients who had previously received cranial irradiation, including whole-brain radiation and non-breast SRS. Figure 1 is a flowchart that depicts the selection process of this analysis.

### 2.3. Data Extraction

Using a specific extraction form, three of the authors independently extracted data from the included articles to ensure accuracy. Any disagreements were resolved by a group discussion with the corresponding author. The following study characteristics were tabulated: author, year of publication, country of origin, retrospective versus prospective, single or multi-center study, treatment modality utilized, chemotherapy, hormone therapy, number of patients, age, tumor volume, number of brain metastases, histology, hormone receptor type, molecular subtype, radiation dose, survival time, local recurrence, distant recurrence, salvage therapy, adverse events, and neurologic death. Progression-free survival was tabulated from individual studies and defined as the time interval between the end of stereotactic surgery and the date of the first CNS progression. Distant failure was defined by the presence of new brain metastases or leptomeningeal enhancement outside the irradiated volume.

### 2.4. Statistical Analysis

Pooled estimates, weighted by sample size, were calculated using either a fixed effects or random effects model based on the DerSimonian and Laird approach. This method accounted for both within-study and between-study variance. To evaluate heterogeneity among studies, we calculated the I^2^ statistic, which measures the proportion of variation due to differences among studies rather than random chance. An I^2^ value greater than 50% was considered indicative of substantial heterogeneity. Forest plots were created to illustrate the prevalence and overall estimated rates, accompanied by 95% confidence intervals (CIs). Due to the single-arm nature of the pooled analyses, funnel plots for assessing publication bias could not be generated. All statistical analyses were performed using the “meta” package in RStudio (https://www.r-project.org/, accessed on 26 August 2024).

### 2.5. PRISMA Statement

The systematic review followed the recommendations of the Preferred Reporting Items for Systematic Reviews and Meta-Analyses (PRISMA). The protocol has not been registered.

## 3. Results

### 3.1. Study Identification and Characteristics

A literature search was conducted of studies using stereotactic radiosurgery to treat intracranial metastases from breast carcinoma, which yielded 29 studies. Reviewing these articles, there were found to be 16 studies that met this meta-analysis inclusion criteria [3,4,14,15,16,17,18,19,20,21,22,23,24,25,26,27]. Table 1 identifies these studies and displays the overall characteristics of each scientific article.

Of these 16 studies, 10 (63%) were found to be retrospective case-control studies, while the remaining 6 (37%) were identified as prospective cohort studies. Twelve (75%) of these studies relied on data from a single institution, with the data for the other four studies coming from multiple institutions. Five studies (32%) were performed in the USA, three (19%) in Germany, two (13%) in Italy, and the remaining six in Slovenia, Turkey, Japan, England, Denmark, and Australia. Combined, the studies treated a total of 3310 lesions with SRS in 1775 patients. A total of 449 patients were treated with chemotherapy, while 131 and 251 patients were treated with hormonal therapy and anti-HER2 therapy, respectively. The methodological quality of the studies was high, which ranged from 7 to 8. The detailed methodological quality assessment is found in Table 2. Details of tumor and treatment characteristics of the included studies are provided in Table 3.

### 3.2. Meta-Analysis of SRS Outcomes

**Figure 2:**
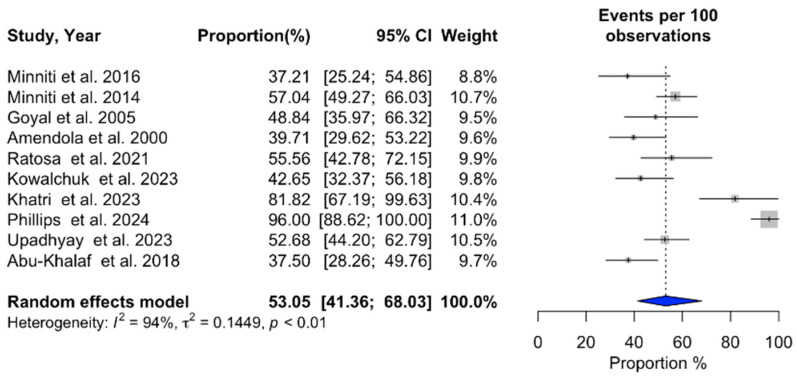
Meta-analysis of 1-year overall survival shown as a forest plot. Plots weighted for sample size show the 1-year overall survival rate ranging from 41.36% to 68.03%. CI, confidence interval [3,14,15,18,19,20,21,22,24,27].

**Figure 3:**
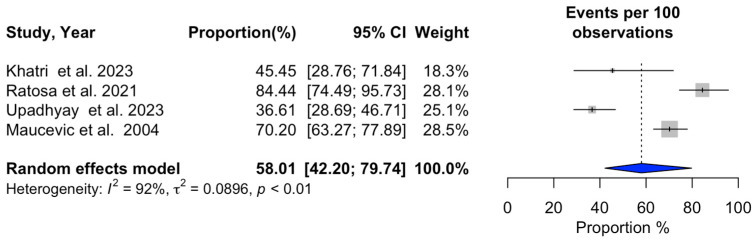
Meta-analysis of 1-year progression-free survival shown as a forest plot. Plots weighted for sample size show the 1-year progression-free survival rate ranging from 42.20% to 79.74%. CI, confidence interval [3,19,23,27].

**Figure 4:**
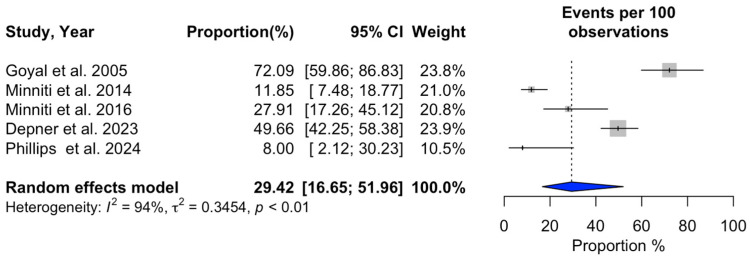
Meta-analysis of 1-year local recurrence rate shown as a forest plot. Plots weighted for sample size show the 1-year local recurrence rate ranging from 16.65% to 51.96%. CI, confidence interval [17,18,21,22,24].

**Figure 5:**
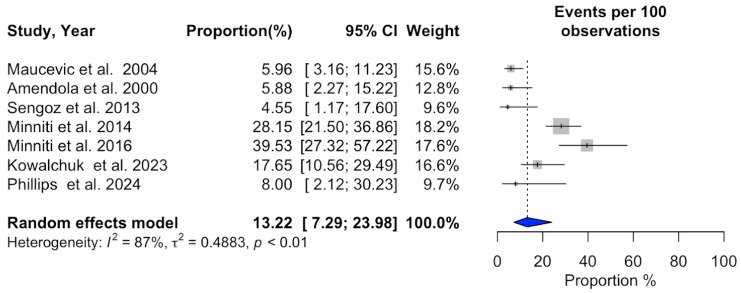
Meta-analysis of overall local recurrence rate shown as a forest plot. Plots weighted for sample size show the overall local recurrence rate ranging from 7.29% to 23.98%. CI, confidence interval [15,20,21,22,23,24,25].

**Figure 6:**
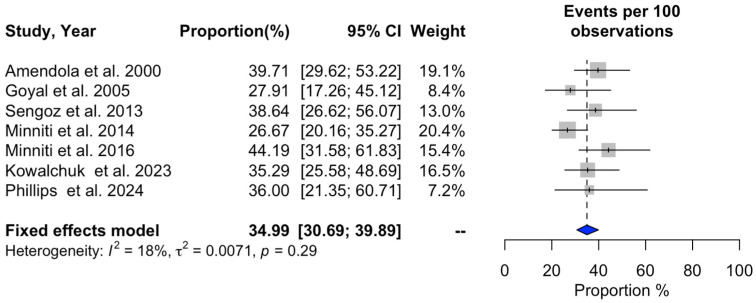
Meta-analysis of overall distant recurrence rate shown as a forest plot. Plots weighted for sample size show the distant recurrence rate ranging from 30.69% to 39.89%. CI, confidence interval [15,18,20,21,22,24,25].

**Figure 7:**
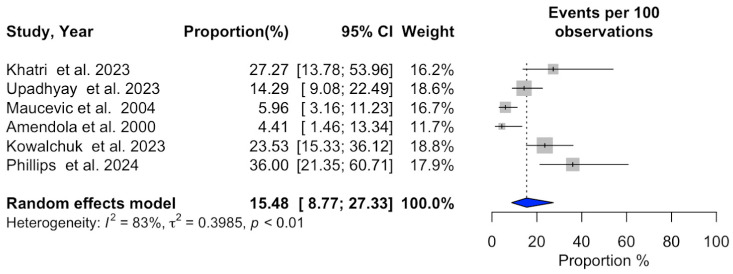
Meta-analysis of acute adverse event rate shown as a forest plot. Plots weighted for sample size show the acute adverse event rate ranging from 8.77% to 27.33%. CI, confidence interval [15,19,20,23,24,27].

**Figure 8:**
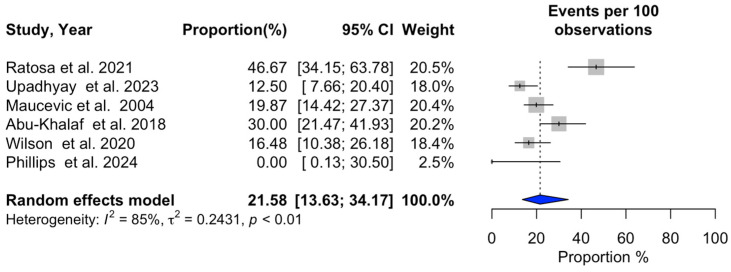
Meta-analysis of neurological death shown as a forest plot. Plots weighted for sample size show the neurological death rate ranging from 13.63% to 34.17%. CI, confidence interval [3,4,14,23,24,27].

Ten studies (n = 641) provided data on 1-year overall survival after SRS (Table 4), revealing a pooled survival rate of 53.05% (95% CI: 41.36–68.03%) [3,14,15,18,19,20,21,22,24,27]. Progression-free survival at 1 year was reported in four studies (n = 330), with a pooled rate of 58.01% (95% CI: 42.20–79.74%) [3,19,23,27]. Local recurrence occurred in 29.42% of patients one year after SRS, based on five studies (n = 395) (95% CI: 16.65–51.96%) [17,18,21,22,24]. The overall local recurrence rate, as reported by seven studies (n = 534), was 13.22% (95% CI: 7.29–23.98%) [15,20,21,22,23,24,25]. Additionally, the overall distant recurrence rate, derived from seven studies, was 34.99% (95% CI: 30.69–39.89%) [15,18,20,21,22,24,25]. Acute adverse events following SRS were documented in six studies (n = 446), with a rate of 15.48% (95% CI: 8.77–27.33%) [15,19,20,23,24,27]. Neurological death occurred in 21.58% of patients, according to six studies (n = 504) (95% CI: 13.63–34.17%) [3,4,14,23,24,27]. Detailed findings are illustrated in Figure 2, Figure 3, Figure 4, Figure 5, Figure 6, Figure 7 and Figure 8.

Significant heterogeneity was observed across studies for 1-year overall survival (I^2^ = 94%, *p* < 0.01), 1-year progression-free survival (I^2^ = 92%, *p* < 0.01), 1-year local recurrence (I^2^ = 94%, *p* < 0.01), overall local recurrence (I^2^ = 87%, *p* < 0.01), acute adverse events (I^2^ = 83%, *p* < 0.01), and neurological death (I^2^ = 85%, *p* < 0.01). In contrast, no substantial heterogeneity was detected in the studies reporting a distant recurrence rate (I^2^ = 18%, *p* = 0.29). Additional information can be found in Figure 2, Figure 3, Figure 4, Figure 5, Figure 6, Figure 7 and Figure 8.

## 4. Discussion

Stereotactic radiosurgery has gained recognition as a pivotal treatment for intracranial metastases, particularly in the context of breast cancer given the relatively higher rates of long-term survival for many patients [3,28]. This approach allows for precise targeting of metastatic lesions, providing a highly conformal radiation dose while minimizing damage to surrounding healthy brain tissue and reducing the cognitive side effects commonly associated with WBRT [3,20]. SRS has demonstrated efficacy in treating small to moderate-sized brain metastases, with studies showing local control rates of 65% to 86% at one year for lesions ≤ 1.5 cm [28,29]. Given the increasing incidence of brain metastases in breast cancer patients, with rates of 7% to 16% at diagnosis and up to 35% during follow-up, understanding the effectiveness of SRS in this population is crucial for optimizing treatment strategies and improving patient outcomes [3,5,30].

Previous studies have shown mixed results regarding the efficacy of SRS for brain metastases across various primary cancer types. Khan et al. conducted a meta-analysis of five randomized controlled trials (n = 763) comparing whole brain radiotherapy (WBRT) alone, SRS alone, and their combination [31]. They found no significant survival benefit for any treatment approach, with a hazard ratio of 1.03 (95% CI: 0.82–1.29, *p* = 0.81) for SRS alone compared to the combined approach. However, they noted that local control was best achieved when WBRT was combined with SRS. Similarly, Stafinski et al. performed a meta-analysis of three randomized controlled trials and one cohort study. They reported no difference in survival between WBRT + SRS and WBRT alone for patients with multiple metastases [32]. However, they observed a statistically significant survival benefit favoring WBRT + SRS in patients with a single metastasis. Additionally, they found significantly higher local tumor control rates at 24 months in the WBRT + SRS treatment arm, regardless of the number of metastases.

In contrast to these general findings across various primary cancer types, our meta-analysis focusing specifically on breast cancer patients with brain metastases revealed more favorable outcomes. We analyzed 16 studies encompassing 1228 patients and observed a trend of improving overall survival from the early 2000s to 2024. Our analysis showed a median survival duration of 13.1 ± 3.8 months and a pooled 1-year overall survival rate of 53.05% after SRS treatment. This trend represents a significant advancement from the early 2000s, when prognosis for patients with brain metastases was markedly poor. Frisk et al. reported a median survival time of only 3 months from the day of first admission for brain metastasis in patients diagnosed between 1998 and 2006, underscoring the limited efficacy of treatments available during that period [33]. These findings suggest that the efficacy of SRS may vary depending on the primary cancer type, with breast cancer patients potentially benefiting more from this targeted treatment modality. Specifically, for triple-negative breast cancer, a subgroup of breast cancer in which overall survival remains poor, brain metastases with this subtype have been shown to respond favorably to SRS [20]. As systemic therapies continue to evolve for triple-negative breast cancer, the high rate of local control that SRS affords to this patient population is paramount to mitigate the worsening of neurological symptoms.

The observed improvement in outcomes for patients with intracranial metastases from primary breast tumor pathology can be attributed to several factors, primarily the advent of targeted therapies and refinements in local treatment modalities. The introduction of trastuzumab (Herceptin) [34], approved in 1998 and widely adopted in the early 2000s, has demonstrated particular efficacy in treating brain metastases, especially when combined with SRS, for HER2-positive breast cancers. Recent studies further corroborate this trend of improved survival. Notably, one study reported that patients with HR-positive/HER2-positive disease exhibited the longest overall survival (median 18 months) among patients with brain metastases at initial breast cancer diagnosis, with 12.2% surviving at 8 years [35]. This marked improvement in long-term survival rates underscores the synergistic impact of advancements in both systemic therapies and local treatments such as SRS.

However, our analysis also highlights the ongoing challenges in treating this patient population. The 29% local recurrence rate at 1 year and 35% overall distant recurrence rate underscore the aggressive nature of metastatic breast cancer and the need for multimodal approaches. It is important to point out that our study specifically focused on brain metastases from breast cancer, whereas much of the existing literature on SRS outcomes includes brain metastases from various primary cancers. This distinction is important, as breast cancer brain metastases may behave differently compared to those from other primary sites. The heterogeneity of breast cancer subtypes further complicates the interpretation of our results. Previous studies have demonstrated that HER2-positive and triple-negative breast cancers have a higher propensity for brain metastases and may respond differently to SRS compared to hormone receptor-positive subtypes [3,7]. For instance, Sperduto et al. reported that HER2-positive patients had a median survival of 24.9 months after brain metastases diagnosis, compared to 8.3 months for triple-negative patients [36]. These subtype-specific differences highlight the need for future studies to investigate the efficacy of SRS based on different molecular subtypes of metastatic breast cancer. Clinicians must be cognizant of not only the efficacy of SRS alone as a treatment modality for intracranial breast metastasis, but also have multidisciplinary discussions to optimize salvage treatments for brain metastases. Future research investigating the potential synergy intracranially between SRS and immune checkpoint inhibition will potentially identify the subset of breast cancer brain metastases who may have an increased benefit of intracranial tumor control from SRS [37].

While WBRT has been a standard approach for multiple brain metastases, its associated cognitive side effects can significantly impact quality of life, especially for long-term survivors [38]. The neurocognitive decline associated with WBRT can be particularly detrimental for breast cancer patients who may live with the effects for years. This consideration has led to a paradigm shift toward SRS, which delivers a highly conformal tumoricidal dose with a reduced risk of cognitive impairment. Our analysis found a relatively low rate of acute adverse events at 15.48% (95% CI: 8.77–27.33%) for SRS, supporting its overall safety and tolerability. This compares favorably with the cognitive side effects often associated with WBRT. The acute adverse events observed in SRS are typically mild and transient, including headaches, nausea, fatigue, and dizziness, which usually resolve within a few days to weeks after treatment [39].

However, it is important to note that late toxicities, such as radiation necrosis, may occur and require surveillance neuroimaging and ongoing monitoring. Radiation necrosis is a serious complication that can develop months to years after SRS, with reported rates ranging from 5% to 25% depending on various factors [39]. The risk of radiation necrosis is related to the volume of brain tissue receiving high doses of radiation, with the volume encompassed by the 12 Gy isodose line being a significant predictor [40]. Despite these potential late effects, the overall safety profile of SRS, particularly in terms of preserving cognitive function, makes it an attractive option for breast cancer patients with brain metastases, who may have longer survival times and thus a higher risk of experiencing long-term cognitive effects from WBRT.

### 4.1. Recommendations for SRS in Breast Cancer Brain Metastases

Current guidelines and emerging data inform recommendations for the use of SRS in breast cancer patients with brain metastases. The American Society for Radiation Oncology guidelines suggest that SRS should be considered for patients with limited brain metastases (generally up to four lesions), good performance status, and well-controlled extracranial disease [41]. For breast cancer specifically, the following factors should be considered when recommending SRS:

(1) Number and size of metastases: SRS is typically recommended for patients with 1–4 brain metastases, each less than 3–4 cm in diameter [42].
(2) Molecular subtype: HER2-positive and triple-negative breast cancers have a higher propensity for brain metastases and may respond differently to SRS. Studies have shown that HER2-positive patients tend to have better outcomes, with median survival times of up to 28 months after brain metastasis diagnosis [43].
(3) Systemic therapy options: the availability of effective systemic therapies, particularly for HER2-positive disease, may influence the decision to use SRS over WBRT, as these patients may have longer survival and thus a higher risk of cognitive effects from WBRT [44].
(4) Performance status: patients with good performance status (KPS ≥ 70) are generally better candidates for SRS.
(5) Extracranial disease control: well-controlled extracranial disease supports the use of SRS, as these patients may have longer overall survival.

Recent data also suggest that SRS may be appropriate for patients with a higher number of brain metastases than previously thought. Some studies have shown comparable outcomes for patients with 5–10 metastases treated with SRS compared to those with 1–4 lesions [45].

While our meta-analysis supports the efficacy and safety of SRS for breast cancer brain metastases, treatment decisions should be individualized based on these factors and made in the context of a multidisciplinary tumor board. Future research should focus on several critical areas to further improve outcomes for breast cancer patients with brain metastases. First, there is a need for studies that explore the optimal timing and sequencing of SRS in conjunction with systemic therapies. Understanding how to best integrate these treatment modalities could enhance therapeutic efficacy and minimize recurrence rates. Additionally, the development of predictive biomarkers for response to SRS could significantly improve patient selection and treatment personalization. Identifying patients who are most likely to benefit from SRS or are at higher risk for adverse effects would allow for tailored treatment plans that maximize efficacy while minimizing toxicity. Long-term follow-up studies are also necessary to assess the durability of treatment responses and the long-term effects of SRS on cognitive function and quality of life. By addressing these areas, future research can contribute to a more comprehensive understanding of the management of brain metastases in breast cancer patients and lead to improved treatment strategies that enhance patient outcomes.

This meta-analysis provides several novel insights that extend beyond the findings of individual studies. Firstly, by aggregating data from 16 studies encompassing 1228 patients, we offer a more comprehensive and statistically robust assessment of SRS outcomes specifically for breast cancer brain metastases. This focus on breast cancer distinguishes our analysis from previous studies that often included mixed primary cancer types. Secondly, our findings reveal a trend of improving overall survival from the early 2000s to 2024, with a median survival duration of 13.1 ± 3.8 months and a pooled 1-year overall survival rate of 53.05% after SRS treatment. This trend highlights the evolving landscape of breast cancer treatment, reflecting both advancements in SRS techniques and the synergistic effects of modern systemic therapies. Thirdly, our analysis of adverse events (15.48%) provides a clearer picture of the risk–benefit profile of SRS in this specific patient population. Lastly, by examining outcomes across different breast cancer subtypes, our study underscores the need for tailored approaches to SRS based on molecular profiles. These insights contribute to a more nuanced understanding of SRS in the context of breast cancer brain metastases and can guide future clinical decision-making and research directions.

### 4.2. Limitations

There are several limitations to acknowledge in this meta-analysis. First, significant heterogeneity was observed across the included studies in measures of survival, recurrence, adverse events, and neurological death. The variations observed can be attributed to several factors, including patient selection criteria, radiation dose and fractionation schemes, disease severity, molecular subtypes of breast cancer, and concurrent systemic therapies. These sources of heterogeneity limit the generalizability of our findings and highlight the need for more standardized reporting and study designs in future research on SRS for breast cancer brain metastases.

Another limitation is the lack of direct comparison between SRS and other treatment modalities, such as WBRT or surgery. We were unable to directly compare the efficacy and safety of SRS with alternative treatments due to the absence of primary studies with such direct comparisons. As a result, while we report a relatively low rate of acute adverse events for SRS, it is challenging to definitively state whether this rate is acceptable without a direct comparison with other treatment modalities. Future studies incorporating head-to-head comparisons between SRS and other treatments are needed to better contextualize the relative benefits and risks of each approach.

## 5. Conclusions

The development of intracranial metastases from breast cancer significantly increases morbidity and mortality, thus posing a challenge for physicians to effectively treat them. Recent advances in SRS have shown promising local tumor control while minimizing neurocognitive side effects. Our study demonstrates that SRS is both effective and safe in managing intracranial metastases from breast cancer, with a favorable side effect profile. Further research is required to determine optimal treatment regimens to provide the most favorable outcomes for such patients.

## Figures and Tables

**Table 1:** Overall Characteristics of the Included Studies.

Author	Year	Country	Study Design	Single Center or Multi-Center?	Sample Size (n)	Treatment Modalities	Chemotherapy (n)	Hormonal Therapy (n)	Anti-HER2 Therapy
Khatri et al. [19]	2023	USA	Retrospective	Single Center	22	SRS and Tucatinib	NA	NA	22
Ratosa et al. [3]	2021	Slovenia	Retrospective	Single Center	45	SRS with or without WBRT	22	15	22
Upadhyay et al. [27]	2023	USA	Retrospective	Single Center	112	SRS	NA	NA	NA
Maucevic et al. [23]	2004	Germany	Prospective	Single Center	151	SRS	113	NA	NA
Amendola et al. [15]	2000	USA	Retrospective	Single Center	68	GKRS alone: 38 GKRS after failed response to WBRT: 30	NA	NA	NA
Combs et al. [16]	2004	Germany	Prospective	Single Center	62	SRS alone: 10 WBRT + SRS focal boost: 13 WBRT + salvage SRS: 39	NA	NA	NA
Goyal et al. [18]	2005	USA	Retrospective	Single Center	43	GKRS + WBRT: 30 GKRS + Surgical Resection: 3	13	NA	NA
Sengoz et al. [25]	2013	Turkey	Retrospective	Single Center	151	GKRS	113	NA	NA
Minniti et al. [21]	2014	Italy	Prospective	Multi-center	125	SRS in 3 × 9 Gy or 3 × 12 Gy	NA	NA	NA
Minniti et al. [22]	2016	Italy	Prospective	Single Center	43	3 × 8 Gy tumors < 2 cm 3 × 7 Gy Tumors ≥ 2 cm	NA	NA	NA
Suzuki et al. [26]	2016	Japan	Retrospective	Single Center	90	GKRS	NA	NA	24
Abu-Khalaf et al. [14]	2019	USA	Retrospective	Single Center	80	GKRS	NA	NA	NA
Wilson et al. [4]	2020	England	Retrospective	Single Center	91	SRS	56	34	34
Kowalchuk et al. [20]	2023	USA	Retrospective	Multi-center	68	GKRS	NA	NA	49
Depner et al. [17]	2023	Denmark	Retrospective	Multi-center	149	SRS	110	73	76
Phillips et al. [24]	2024	Australia	Prospective	Multi-center	25	Surgical Resection + SRS	22	9	24

**Table 2:** Quality Assessment.

First Author	Case-Control or Cohort Studies	S1	S2	S3	S4	C	O1	O2	O3	NOS Scores
Khatri et al. [19]	Case-control	a*	a*	a*	a*	*	b*	a*	b*	8 Stars
Ratosa et al. [3]	Case-control	a*	c	a*	a*	*	a*	a*	b*	7 Stars
Upadhyay et al. [27]	Case-control	a*	a*	b*	a*	*	a*	a*	a*	8 Stars
Maucevic et al. [23]	Cohort	a*	a*	a*	a*	*	a*	a*	a*	8 Stars
Amendola et al. [15]	Case-control	a*	c	a*	a*	*	a*	a*	b*	7 Stars
Combs et al. [16]	Cohort	a*	a*	a*	a*	*	b*	a*	b*	8 Stars
Goyal et al. [18]	Case-control	a*	a*	a*	a*	*	a*	a*	b*	8 Stars
Sengoz et al. [25]	Case-control	a*	a*	a*	a*	*	b*	a*	b*	8 Stars
Minniti et al. (2014) [21]	Cohort	a*	a*	a*	a*	*	a*	a*	b*	8 Stars
Minniti et al. (2016) [22]	Cohort	a*	a*	a*	a*	*	a*	a*	b*	8 Stars
Suzuki et al. [26]	Case-control	b*	a*	b*	a*	*	b*	a*	b*	8 Stars
Abu-Khalaf et al. [14]	Case-control	a*	a*	a*	a*	*	a*	a*	a*	8 Stars
Wilson et al. [4]	Case-control	a*	a*	b*	a*	*	b*	a*	a*	8 Stars
Kowalchuk et al. [20]	Case-control	a*	c	a*	a*	*	a*	a*	b*	7 Stars
Depner et al. [17]	Case-control	a*	c	a*	a*	*	b*	a*	b*	8 Stars
Phillips et al. [24]	Cohort	a*	a*	a*	a*	*	b*	b*	a*	7 Stars

The a, b, c, and * are derived from the coding manual for Newcastle-Ottawa Scale (NOS) to assess quality of both case control and cohort studies (https://www.ohri.ca/programs/clinical_epidemiology/oxford.asp, accessed on 26 August 2024).

**Table 3:** Tumor and Treatment Characteristics of the Included Studies.

First Author	Age (Range)	Tumor Diameters (cm)	Tumor Volume (cm^3^)	Number of Brain Metastases	Histology (Type and Number)	Hormone Receptor (Type and Number)	Molecular Subtype (Type and Number)	Radiation Dose (Gy)/ Fractionation Scheme
Khatri et al. [19]	50.7 (32–66)	NA	0.15 (0–26.89)	125	NA	HER2+ (22, all patients)		SRS: Median of 24 Gy (range: 16–24) fSRS: Median of 27 Gy (range: 20–50)
Ratosa et al. [3]	57 (48–67)	NA	0.11	315	All patients triple-negative breast cancer	ER+: 24 ER−: 21 PR+: 20 PR−: 25 HER2+: 26 HER2−: 17	TNBC: 9 Luminal A: 4 Luminal B HER2−: 6 Luminal B HER2+: 14	18 Gy
Upadhyay et al. [27]	55 (31–84)	NA	5.7	186	NA	ER/PR+ and HER2−: 37 ER/PR− and HER2+: 20 ER/PR+ and HER2+: 24 ER/PR−and HER2−: 31	TNBC: 31 Luminal A: 37 Luminal B: 24 HER-2: 20 Basal: 32	Fractionated SRS: 87 Single Fraction SRS: 25
Maucevic et al. [23]	60 (35–78)	NA	2.2 (0.1–20.9)	620	Infiltrating ductal or lobular carcinoma	NA	NA	19 +/− 4 Gy
Amendola et al. [15]	52 (25–80)	NA	3.3	518	Breast carcinoma	NA	NA	Single Fraction SRS, 6–24 Gy
Combs et al. [16]	<40 years: n = 19 ≥40 years: n = 43	<1 cm: n = 23 1–2 cm: n = 62 2–4 cm: n = 16 ≥4 cm: n = 2	NA	103	Breast Carcinoma	NA	NA	group 1: single dose between 15 and 20 Gy group 2: WBRT with 30–40 Gy using conventional fractionation
Goyal et al. [18]	48 (32–92)	NA	4.1	84	Breast carcinoma	NA	NA	max: 51.17 Gy min: 17.9 Gy
Sengoz et al. [25]	57 (42–82)	Not Recorded	0.6 (Range: 0.34–7.3)	46	Reported Primary Locations: Lung: 28 Breast: 7 Colon: 3 Renal Cell Carcinoma: 3 Others: 3	NA	NA	Not Mentioned
Minniti et al. (2014) [21]	61 (32–79)	Not Recorded	10.1 (Range: 1.6–48.4)	171 Lesions	Lung carcinoma: 65 Breast carcinoma: 32 Melanoma: 19 renal cell carcinoma: 7 Colon Carcinoma: 5 Other: 7	NA	NA	SRS in 3 × 9 Gy or 3 × 12 Gy
Minniti et al. (2016) [22]	61	20 < 2 cm 23 ≥ 2 cm	12.3 (range: 1.5–33.1)	Single Met.: 18 Multiple Mets.: 25 47 Total Lesions	NSCLC: 17 Breast Carcinoma: 9 Melanoma: 11 Other: 6	NA	NA	7–8 Gy in 3 daily fractions
Suzuki et al. [26]	53 (30–80)	NA	0.25	623	NA	NA	HER2-targeting agents used: 22	single fraction median dose 18 Gy
Abu-Khalaf et al. [14]	56.2	NA	NA	NA	Breast carcinoma	ER+: 38 ER−: 38 ER unknown: 4 PR+: 28 PR−: 46 PR unknown: 6 HER2+: 38 HER2−: 33 HER2 unknown: 6	luminal A: 16 luminal B: 19 HER2: 17 Basal: 17 Unknown: 11	16–24 Gy single fraction
Wilson et al. [4]	57 (23–78)	NA	3.8 (0.1–24.5)	1 lesion: n = 39 2–5 lesions: n = 33 ≥6 lesions: 19	NA	ER+/HER2−: 31 ER+/HER2+: 14 ER−/HER2+: 30	TNBC: 16 Luminal A: 31 Luminal B: 14 HER-2: 30	<2 cm: 24 Gy 2–3 cm: 22 Gy 3–4 cm: 20 Gy single fraction
Kowalchuk et al. [20]	57 (48–67)	NA	0.11	315	triple-negative breast cancer	NA	TNBC: 68	18 Gy
Depner et al. [17]	59	NA	3.73	267	IDC- 131 ILC- 6 OTHER- 12	ER+/HER2+: 39 ER+/HER2−: 49 ER−/HER2+: 37 ER−/HER2−: 22	Lumina A: 49 Lumina B: 39 HER-2: 37 Basal: 22	18 Gy delivered in 1–3 fractions
Phillips et al. [24]	57.3 (13.2)	NA	0.6 (0.0–14.4)	42	Metastatic HER2+ breast cancer: 25	NA	NA	18 Gy in 1 fraction: 3 20 Gy in 1 fraction: 24 22 Gy in 1 fraction: 4 24 Gy in 3 fractions: 9 30 Gy in 5 fractions: 1

**Table 4:** SRS Outcomes.

First Author	Median Survival Time (Months)	1-Year Overall Survival (n)	1-Year Progression Free Survival (n)	1-Year Local Recurrence (n)	Overall Local Recurrence (n)	Distant Recurrence (n)	Salvage Therapy	Acute Adverse Events (n)	Please Specify	Late Adverse Events (n)	Please Specify	Neurological Death (n)
Khatri et al. [19]	21.2	18	11	1	4	16	NA	6	Symptomatic Radiation Necrosis	NA	NA	NA
Ratosa et al. [3]	18.5	25	38	NA	NA	NA	NA	17	Radiographic Leptomeningeal Carcinomatosis	NA	NA	21
Upadhyay et al. [27]	13.1	59	41	NA	NA	NA	WBRT	16	Radiation Necrosis	NA	NA	14
Maucevic et al. [23]	10	NA	106	NA	9	42	SRS	9	Leptomeningeal Disease	NA	NA	30
Amendola et al. [15]	7.8	27	NA	NA	4	27	GKS	3	Brain necrosis	NA	NA	NA
Combs et al. [16]	15	NA	NA	NA	NA	NA	SRS: 11 patients	NA	NA	NA	NA	NA
Goyal et al. [18]	13	21	NA	31	NA	12	NA	NA	NA	NA	NA	NA
Sengoz et al. [25]	8	NA	NA	NA	2	17 Brain Mets	SRS after WBRT as a salvage treatment: 23 WBRT+SRS+ WBRT: 3 WBRT after SRS: 6 SRS only: 15	8	NA	NA	NA	2
Minniti et al. (2014) [21]	10	16	NA	12	17	19	WBRT (n = 6), SRS (n = 14), or both (n = 3)	0	NA	15	Radionecrosis: 9 (4 via biopsy) Grade 3 motor deficits: 3 Grade 2 speech deficits: 1 Grade 2 confusion: 2)	NA
Minniti et al. (2016) [22]	14.8	77	NA	16	38	36	WBRT: 27 Further SRS: 34	0	NA	20	Radionecrosis: 12 Saizure: 4 Motor deficits: 2 Confusion: 2 Speech defecits: 2	NA
Suzuki et al. [26]	10.8	NA	NA	NA	NA	NA	NA	NA	NA	NA	NA	NA
Abu-Khalaf et al. [14]	13.1	30	NA	NA	NA	NA	14	NA	NA	NA	NA	24
Wilson et al. [4]	15.7	NA	NA	NA	NA	NA	NA	NA	NA	NA	NA	15
Kowalchuk et al.	10	29	NA	NA	12	24	Additional SRS: 7 Chemotherapy: 10	16	Radiation necrosis: 8 Leptomeningeal disease: 6 New cranial nerve deficit: 2	NA	NA	NA
Depner et al. [17]	14.8	NA	NA	74	NA	NA	NA	NA	NA	96	not specified	NA
Phillips et al. [24]	NA	24	NA	2	2	9	NA	29	allergic skin reaction: 1 seizure: 1 headache: 11 fatigue: 16	14	memory impairment: 3 other: 11	NA

## Data Availability

The original contributions presented in the study are included in the article, further inquiries can be directed to the corresponding author.
